# Supplementary material for: A clonally expanded nodal T-cell population diagnosed as T-cell lymphoma after CAR-T therapy
Source: Nat Commun. 2025 Aug 12;16:7462. doi: 10.1038/s41467-025-62709-7 (PMC12343882; doi:10.1038/s41467-025-62709-7)
Supplement: Supplementary file 1 — Supplementary Information [file 41467_2025_62709_MOESM1_ESM.pdf]

A clonally expanded nodal T-cell population diagnosed as T-cell lymphoma after CAR-T therapy

Katie Maurer[1,2,3]*, Jackson A. Weir[3,4]*, Adi Nagler[1,2,3], Nicholas J. Haradhvala[3], Hariharan Bharadwaj[5], Jacob Shapiro[3,6], Somkene Alakwe[3], Vipin Kumar[3], Brianna Waller[5], Mikaela McDonough[1], Jamie Dela Cruz[1], Loida Luna[1], Emma Lin[1], Linsey Gong[3], Qiyu Gong[3], Mehdi Borji[3], Phillip D. Michaels[5], Jacob P. Laubach[1,2], Geraldine Pinkus[5], Gad Getz[3], Catherine J. Wu[1,2,3†], Fei Chen[3†], Caron Jacobson[1,2,†]

Supplementary Figure

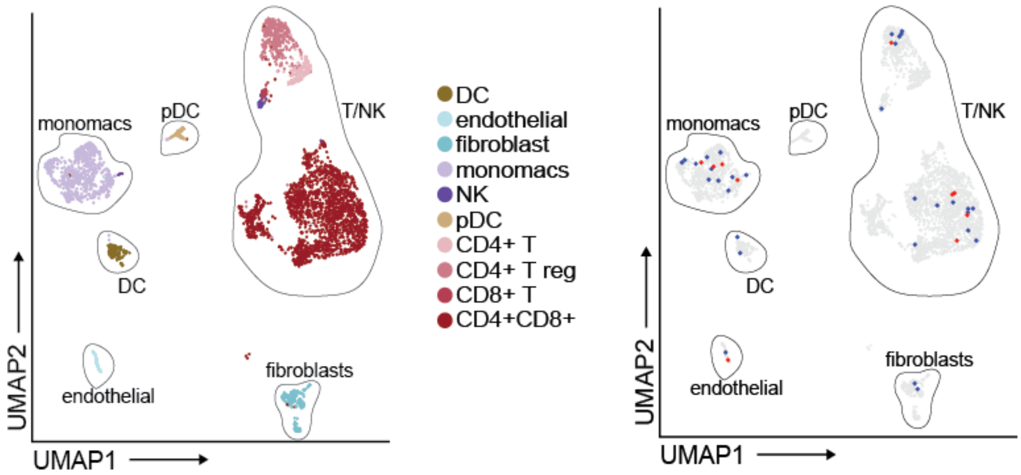

Supplementary Fig. 1 Legend: Left UMAP - all cells annotated by major cell type, reproduced from Figure 3A; Right UMAP - cells in which the wild-type (blue) or mutant (red) *TET2* p.776Wfs*4 sequences are identified. Cells in which neither wild type nor mutant sequence is identified are shown in gray.
