# Supplementary material for: A clonally expanded nodal T-cell population diagnosed as T-cell lymphoma after CAR-T therapy
Source: Nat Commun. 2025 Aug 12;16:7462. doi: 10.1038/s41467-025-62709-7 (PMC12343882; doi:10.1038/s41467-025-62709-7)
Supplement: Supplementary file 2 — Description of Additional Supplementary Files [file 41467_2025_62709_MOESM2_ESM.pdf]

Description of Additional Supplementary Files

Supplementary Data 1. MiXCR analysis of TCR data from WGS

Supplementary Data 2. Nonsilent variants detected in lymph node WGS.

Supplementary Data 3. Top 100 genes correlated with each T-cell gene expression program score ranked by Pearson correlation.

Supplementary Data 4. Cycle thresholds for CAR transcript qPCR.

Supplementary Data 5. Counts of TCR alpha and beta chains in the Slide-tags 5’ snRNA-seq data.

Supplementary Data 6. Public TCR clonotypes.
