# Supplementary material for: A clonally expanded nodal T-cell population diagnosed as T-cell lymphoma after CAR-T therapy
Source: Nat Commun. 2025 Aug 12;16:7462. doi: 10.1038/s41467-025-62709-7 (PMC12343882; doi:10.1038/s41467-025-62709-7)
Supplement: Supplementary file 4 — Reporting Summary [file 41467_2025_62709_MOESM4_ESM.pdf]

nature portfolio

Corresponding author(s): *Double-anonymous peer review submissions: write DAPR and your manuscript number here instead of author names.*

Last updated by author(s): *YYYY-MM-DD*

Reporting Summary

Nature Portfolio wishes to improve the reproducibility of the work that we publish. This form provides structure for consistency and transparency in reporting. For further information on Nature Portfolio policies, see our Editorial Policies and the Editorial Policy Checklist.

Statistics

For all statistical analyses, confirm that the following items are present in the figure legend, table legend, main text, or Methods section.

n/a	Confirmed	
☒	☐	The exact sample size (*n*) for each experimental group/condition, given as a discrete number and unit of measurement
☒	☐	A statement on whether measurements were taken from distinct samples or whether the same sample was measured repeatedly
☒	☐	The statistical test(s) used AND whether they are one- or two-sided<br>*Only common tests should be described solely by name; describe more complex techniques in the Methods section.*
☒	☐	A description of all covariates tested
☒	☐	A description of any assumptions or corrections, such as tests of normality and adjustment for multiple comparisons
☒	☐	A full description of the statistical parameters including central tendency (e.g. means) or other basic estimates (e.g. regression coefficient) AND variation (e.g. standard deviation) or associated estimates of uncertainty (e.g. confidence intervals)
☒	☐	For null hypothesis testing, the test statistic (e.g. *F*, *t*, *r*) with confidence intervals, effect sizes, degrees of freedom and *P* value noted<br>*Give P values as exact values whenever suitable.*
☒	☐	For Bayesian analysis, information on the choice of priors and Markov chain Monte Carlo settings
☒	☐	For hierarchical and complex designs, identification of the appropriate level for tests and full reporting of outcomes
☒	☐	Estimates of effect sizes (e.g. Cohen's *d*, Pearson's *r*), indicating how they were calculated

Our web collection on statistics for biologists contains articles on many of the points above.

Software and code

Policy information about availability of computer code

Data collection	*Provide a description of all commercial, open source and custom code used to collect the data in this study, specifying the version used OR state that no software was used.*
Data analysis	*Provide a description of all commercial, open source and custom code used to analyse the data in this study, specifying the version used OR state that no software was used.*

For manuscripts utilizing custom algorithms or software that are central to the research but not yet described in published literature, software must be made available to editors and reviewers. We strongly encourage code deposition in a community repository (e.g. GitHub). See the Nature Portfolio guidelines for submitting code & software for further information.

Data

Policy information about availability of data

All manuscripts must include a data availability statement. This statement should provide the following information, where applicable:

- Accession codes, unique identifiers, or web links for publicly available datasets
- A description of any restrictions on data availability
- For clinical datasets or third party data, please ensure that the statement adheres to our policy

Data availability
Single cell transcriptome and TCR data will be submitted to NCBI's Database of Genotypes and Phenotype (dbGaP; https://www.ncbi.nlm.nih.gov/gap) under

accession code TBD and in the National Center for Biotechnology Information's Gene Expression Omnibus upon publication under accession code TBD. WGS data will be submitted to dbGaP under accession code TBD. Source data are provided with this paper.

Code Availability
Code for processing spatial sequencing libraries is available at GitHub (https://github.com/broadchenf/Slide-tags).
Data availability
Single cell transcriptome and TCR data will be submitted to NCBI's Database of Genotypes and Phenotype (dbGaP; https://www.ncbi.nlm.nih.gov/gap) under accession code TBD and in the National Center for Biotechnology Information's Gene Expression Omnibus upon publication under accession code TBD. WGS data will be submitted to dbGaP under accession code TBD. Source data are provided with this paper.

Code Availability
Code for processing spatial sequencing libraries is available at GitHub (https://github.com/broadchenf/Slide-tags).

Research involving human participants, their data, or biological material

Policy information about studies with human participants or human data. See also policy information about sex, gender (identity/presentation), and sexual orientation and race, ethnicity and racism.

Reporting on sex and gender	Sex and gender were not considered in study design, as this was a retrospective analysis of a clinical cohort, and neither sex nor gender were anticipated to play a role in the central findings.
Reporting on race, ethnicity, or other socially relevant groupings	Race, ethnicity, or other socially relevant groupings were not applicable to this retrospective cohort study.
Population characteristics	See above.
Recruitment	This was a retrospective study performed on all patients receiving standard of care CAR-T (i.e. not on clinical trial) therapy at the Dana-Farber Cancer Institute
Ethics oversight	Clinical data and biological samples were collected on study protocols which were approved by the Dana-Farber Cancer Institute/Harvard Cancer Center Institutional Review Board. All patients gave written informed consent to this protocol.

Note that full information on the approval of the study protocol must also be provided in the manuscript.

Field-specific reporting

Please select the one below that is the best fit for your research. If you are not sure, read the appropriate sections before making your selection.

☒ Life sciences ☐ Behavioural & social sciences ☐ Ecological, evolutionary & environmental sciences

For a reference copy of the document with all sections, see nature.com/documents/nr-reporting-summary-flat.pdf

Life sciences study design

All studies must disclose on these points even when the disclosure is negative.

Sample size	The entire cohort of patients receiving standard of care CAR-T therapy was included. No statistical determination of sample size was indicated.
Data exclusions	No data were excluded.
Replication	As this was a retrospective cohort study with a case report, no attempt at reproducibility was possible.
Randomization	As this was a retrospective cohort study with a case report, randomization was not relevant.
Blinding	As this was a retrospective cohort study with a case report, blinding was not relevant.

Reporting for specific materials, systems and methods

We require information from authors about some types of materials, experimental systems and methods used in many studies. Here, indicate whether each material, system or method listed is relevant to your study. If you are not sure if a list item applies to your research, read the appropriate section before selecting a response.

Materials & experimental systems

n/a	Involved in the study
☒	☐ Antibodies
☒	☐ Eukaryotic cell lines
☒	☐ Palaeontology and archaeology
☒	☐ Animals and other organisms
☐	☒ Clinical data
☒	☐ Dual use research of concern
☒	☐ Plants

Methods

n/a	Involved in the study
☒	☐ ChIP-seq
☒	☐ Flow cytometry
☒	☐ MRI-based neuroimaging

Clinical data

Policy information about clinical studies

All manuscripts should comply with the ICMJE guidelines for publication of clinical research and a completed CONSORT checklist must be included with all submissions.

Clinical trial registration: This was not a clinical trial.

Study protocol: Study protocol #17-561 approved by the Dana-Farber/Harvard Cancer Center Institutional Review Board

Data collection: Clinical data was collected from the electronic medical record and a clinical RedCap database on all patients receiving standard of care CAR-T therapy at DFCI between 2017-2023 with a focus on possible post-CAR-T lymphoma.

Outcomes: Outcome of interest was development of post-CAR-T T cell lymphoma, which was determined clinically/pathologically

Plants

Seed stocks: N/A

Novel plant genotypes: N/A

Authentication: N/A
